# Utility of internally transcribed spacer region of rDNA (ITS) and *β*‐tubulin gene sequences to infer genetic diversity and migration patterns of *Colletotrichum truncatum* infecting *Capsicum* spp.

**DOI:** 10.1002/ece3.1918

**Published:** 2016-01-09

**Authors:** Kandyce Rampersad, Hema Ramdial, Sephra N. Rampersad

**Affiliations:** ^1^ Department of Pre‐clinical ScienceFaculty of Medical SciencesThe University of the West IndiesSt. AugustineTrinidad and TobagoWest Indies; ^2^ Department of Life SciencesFaculty of Science and TechnologyThe University of the West IndiesSt. AugustineTrinidad and TobagoWest Indies

**Keywords:** Genetic diversity, migration, pathogenic fungi, population genetics

## Abstract

Anthracnose is among the most economically important diseases affecting pepper (*Capsicum* spp.) production in the tropics and subtropics. Of the three species of *Colletotrichum* implicated as causal agents of pepper anthracnose, *C. truncatum* is considered to be the most destructive in agro‐ecosystems worldwide. However, the genetic variation and the migration potential of *C. truncatum* infecting pepper are not known. Five populations were selected for study and a two‐locus (internally transcribed spacer region, ITS1‐5.8S‐ITS2, and *β*‐tubulin, *β*‐TUB) sequence data set was generated and used in the analyses. Sequences of the ITS region were less informative than *β* **‐**tubulin gene sequences based on comparisons of DNA polymorphism indices. Trinidad had the highest genetic diversity and also had the largest effective population size in pairwise comparisons with the other populations. The Trinidad population also demonstrated significant genetic differentiation from the other populations. AMOVA and STRUCTURE analyses both suggested significant genetic variation within populations more so than among populations. A consensus Maximum Likelihood tree based on *β*‐TUB gene sequences revealed very little intraspecific diversity for all isolates except for Trinidad. Two clades consisting solely of Trinidad isolates may have diverged earlier than the other isolates. There was also evidence of directional migration among the five populations. These findings may have a direct impact on the development of integrated disease management strategies to control *C. truncatum* infection in pepper.

## Introduction

*Colletotrichum* species are among the most destructive plant pathogens worldwide. Member species cause the disease anthracnose, which affects fruit and plant parts in a range of economically important host species including legumes, fruit trees, and vegetables (Bailey et al. 1992).The disease is a constraint to pepper (*Capsicum* spp.) production in the tropics and subtropics (Than et al. 2008). Typical symptoms of anthracnose disease affecting pepper fruit may include sunken necrotic lesions with concentric rings of acervuli within them. In some cases, the lesions are brown, which later turn black after the pathogen forms setae and sclerotia (Roberts et al. 2001). However, the symptoms are less evident on stem and leaves (Alexander and Pernezny 2003).

At least three species of *Colletotrichum*,* C. truncatum* (Schwein.) Andrus & Moore, *C. gloeosporioides* (Penz.) Penz. and Sacc., and *C. acutatum* (Simmonds), have been implicated as important pathogens causing pepper anthracnose, with *C. truncatum* as the most destructive (Sharma et al. 2005; Montri et al. 2009). The truncatum clade includes only one major species, *C. truncatum* (syn. *C. capsici*; (Syd.) Butler and Bisby‐(Damm et al. 2009), which is reported to be a destructive pathogen of many tropical crops including legumes and solanaceous plants.

Different *Colletotrichum* species may demonstrate differential infection depending on the stage of maturity of the pepper fruit. For example, *C*. *truncatum* usually infect red pepper fruits, but *C*. *acutatum* and *C*. *gloeosporioides* are more commonly found as pathogens of both young and mature green fruits (Harp et al. 2008, 2013). Infection may occur as both pre‐ and postharvest fruit rots, which result in considerable reduction of marketable yield. Maximum losses in marketable yield have been reported as 60–80% in countries such as Thailand (Mustafa et al. 2006; Poonpolgul and Kumphai 2007), Malaysia (Mah 1987; Mahmodi et al. 2014), India (Sharma et al. 2005), the U.S. (Harp et al. 2008; Lewis Ivey et al. 2004), and Trinidad (Ramdial and Rampersad 2014). Production and export of peppers in Trinidad fluctuated over the period 2006–2010; however, there was a marked increase in imports from the USA from 62 metric tons in 2006 to 202 metric tons in 2010 (Mohammed 2013).

Survival and dispersal of *Colletotrichum* spp. can occur in and on seeds as acervuli and microsclerotia (Mordue 1971; Meon and Nik 1988) as well as in diseased fruit, which can act as a source of inoculum to infect other crops via water splash or irrigation water (Roberts et al. 2001).The pathogen infects the seed coat (Meon and Nik 1988; Chitkara et al. 1990) and the endosperm tissue in seeds. Hence, the use of infected pepper seeds can give rise to seedlings that serve as a primary inoculum source in the field (Meon and Nik 1988; Pring et al. 1995).

*Capsicum* is one of the oldest cultivated crops of the Americas and has ancestral origins in Central, South America and the Caribbean (DeWitt and Gerlach 1990).The ~30 species of *Capsicum* are all native to the Americas, with a recent consensus model indicating that *C. annuum* originated in Mexico (Kraft et al. 2014). It was domesticated by prehistoric peoples in different parts of Central America (Bosland 1996). The fruit of *Capsicum* is called by a variety of names, but “chile” is a variation of the Aztec dialect for all plants known to be *Capsicum* and “aji” is a variation of “axi” derived from the now‐extinct Arawak dialect of the Caribbean (DeWitt and Gerlach 1990). Of the five species of cultivated *Capsicum, C*. *annuum* is one of the most popularly cultivated crops worldwide. Bell pepper refers to the nonpungent blocky chile type (DeWitt and Gerlach 1990). Christopher Columbus is credited with introducing peppers to Europe, Africa, and Asia; it then spread rapidly through Europe to India, China, and Japan (Bosland 1996). It is also proposed that the Portuguese introduced peppers through their trading activities with Africa, India, and Asia (Wallin 2004).

Management of *Colletotrichum* diseases involves the use of integrated management strategies in addition to screening and breeding for cultivars with intrinsic resistance (Yoon and Park 2001; Yoon et al. 2004). However, information on the degree and distribution of genetic variation within and between populations of *C. truncatum* infecting pepper in different global geographic regions would enable more focused and perhaps shared strategies of control (McDonald and Linde 2002). These populations would have shared genetic signatures that can persist for several thousands of years and, therefore, would provide information about this species' demographic history and changes in its distribution through the course of evolutionary time. Furthermore, host–pathogen association studies reveal that the demographic history of the pathogen coincides with that of the host (Falush et al. 2003).

Genetic markers have been used to investigate population dynamics and migration patterns of plant pathogens at wide‐ranging spatial scales, that is, local movement among fields of a given location to global dispersal (Banke and McDonald 2005). The possible centers of diversity and patterns of migration can be determined by assessing the distribution of genetic diversity within and among populations (Beerli and Felsenstein 2001). The center of origin of a pathogen coincides with populations that demonstrate higher genetic variability than newer, more recently founded populations (Templeton et al. 1995). It is also hypothesized that the pathogen population nearest to the center of origin of the host would (1) represent the oldest population, (2) have the highest levels of genetic diversity, and (3) be highly differentiated from all other populations. Anthropogenic dispersal of infected seed over long distances, for example, through trade, may contribute significantly to observed patterns of genetic variability among pathogen populations (Baker and Smith 1966). Long‐distance seed dispersal may provide opportunities for a mix of contemporary and historical migration events across countries, suggestive of spatial connectivity despite geography (Stukenbrock et al. 2006).

The main objectives of the study were (1) to assess the diversity and phylogenetic relationships of *C. truncatum* infecting pepper, (2) to determine the contribution of migration events to the observed genetic variation, and (3) to identify the relative contribution of sexual and asexual reproduction to pathogen evolution.

## Materials and Methods

### Collection and maintenance of isolates

Bell pepper fields in the main production areas in North and South Trinidad were surveyed at their harvesting stage during the period November 2010 to April 2014. Single‐spore cultures (*N *=* *88) of *C. truncatum* were prepared from symptomatic fruit as described by Ramdial and Rampersad (2014).

### DNA extraction, PCR, and sequencing

Fungal genomic DNA was extracted from mycelia mats using the E.Z.N.A. DNA extraction kit (Omega Bio‐tek, Inc., Norcross, GA). PCR amplification was carried out using the universal primer pair, ITS4/5 (internally transcribed spacer region of the nuclear ITS1‐5.8S‐ITS2 rDNA (White et al. 1990), and TUB1/2 (partial *β*‐tubulin gene; Glass and Donaldson 1995) as described elsewhere (Ramdial and Rampersad 2014). PCR products were sequenced directly in both directions (Amplicon Express, Pullman, WA).

Sequences were identified through comparisons of cognate sequences available in the GenBank and EMBL public databases were made using the gapped BLAST algorithm. Additionally, sequences were also included based on the work of Ramdial and Rampersad (2014). Sequences of representative isolates were deposited in GenBank (GenBank Accession Nos.: KJ780718, GenBank: JF749808, GenBank: HQ287583, GenBank: HQ287585). A total of 98 sequences were used in the final two‐locus data set. For each gene: 10 sequences from the Trinidad population; 8 sequences from the USA population; 7 sequences from the India population; 14 sequences from the Thailand population; 12 sequences from the Malaysia population (Table S1A and B). The USA population was specifically selected for the study because the host species for this population did not include *Capsicum* spp. and was, therefore, used for comparison as a non‐*Capsicum* host population. Only published sequences were included in this study.

### Sequence alignments

Multiple nucleotide sequence alignments were conducted using the online version of the multiple sequence alignment program, MAFFT version 6 (http://mafft.cbrc.jp/alignment/server/). Bioedit sequence alignment editor software version 7.2.5 (http://www.mbio.ncsu.edu/bioedit/page2.html) was used to visually inspect and edit the aligned sequences to common nucleotide lengths for *β*‐TUB and ITS gene regions.

### Genetic variation

DNA polymorphism analyses were performed using DNA Sequence Polymorphism software (DnaSP) version 5.10 (Rozas et al. 2003; Librado and Rozas 2009) and ARLEQUIN version 3.1 (Excoffier et al. 2005). The isolates were categorized into populations based on country of origin of a particular isolate. DNA divergence as the average number of nucleotide differences (*k*), and the nucleotide diversity (*π*) were determined. Watterson's estimator of mutation rate (*θ*‐W) on a per‐sequence basis was also calculated for each population.

The degree of genetic differentiation between populations (*F*_ST_) was determined using DnaSP and ARLEQUIN. Hierarchical analysis of molecular variance (AMOVA) was carried out to assess the proportion of observed variation that was attributable to within‐population differences versus among‐population differences. Significance was assessed using 9999 permutations of the original data using GenAlEx version 6.3 (Peakall and Smouse 2006). Population differentiation as a result of isolation‐by‐distance was estimated based on Mantel's test using GenAlEx with the assumption of a linear relationship between genetic and geographic distances between populations.

Hierarchical analysis of molecular variance (AMOVA) was carried out to assess the proportion of observed variation that was attributable to within‐population differences versus among‐population differences. The significance of these differences was assessed using 9999 permutations of the original data using GenAlEx version 6.3 (Peakall and Smouse 2006).

In silico PCR‐RFLP analysis was carried out to produce a binary matrix to run STRUCTURE and GenAleX to detect the possibility of shared variation among populations. Virtual digests were carried out using Restriction Enzyme Picker Online, v.1.3 (http://rocaplab.ocean.washington.edu/tools/repk), which assisted in selecting specific restriction enzymes that uniquely discriminate the different sequence groups and using NEBCutter V.2 (Vincze et al. 2003) and WEB‐Cutter V.2 (http://bio.lundberg.gu.se/cutter2) based on the approach by Ramdeen and Rampersad (2012). Eighteen restriction enzymes were screened: ApoI, AluI, Ban II, BsaJI, Bsp12861, CviAII, DpnI, EcoRI, FatI, HaeIII, HincII, HinfI, Hpy99I, HpyCH4V, MseI, MspI, Sac II, and SAUIIIA, which resulted in 184 polymorphic loci. A binary matrix was prepared and an assignment test based on Bayesian posterior probability implemented in STRUCTURE version 2.3.2.1 (Pritchard et al. 2000; Falush et al. 2003, 2007) was used to determine the nature of subpopulation structuring. Ten runs with a burn‐in period of 50,000 generations and 100,000 MCMC (Markov chain Monte Carlo) iterations from *K *=* *1 to *K *=* *5; the model does not identify *K *=* *1. This program calculates the membership coefficients to each of the populations (*Q)* of every sample (Falush et al. 2007) and only coefficients >75–80 were considered. At least 20 replicates were used to determine likelihood LnP(D) of population assignment and membership to ensure reproducibility of the results (Gilbert et al. 2012).

### Linkage disequilibrium (LD)

The LD test is useful to determine whether populations are clonal (where significant disequilibrium is expected due to linkage among loci) or sexually reproducing (where linkage among loci is not expected). The extent of linkage disequilibrium among the five populations was determined by detecting allelic association at different loci (Maynard‐Smith et al. 1993). Linkage disequilibrium (LD) (Lewontin 1995) was determined as the number of significant pairwise comparisons based on Fishers' exact test (*P *≤* *0.001) and using Bonferroni correction (*P *≤* *0.001). The Index of Association (*I*_*A*_) and rBarD were also calculated using Multilocus 1.3b (Agapow and Burt 2001) for a separate multilocus data set generated by in silico restriction digestion. The Index of Association (I_A_) and rBarD are expected to be zero if populations are freely recombining and greater than zero if there is association between alleles (clonality). Detection of possible recombination events was also performed using Recombination Detection Program, RDP3.44 (Martin et al. 2010).

### Tests for migration

Migration between pairs of populations was estimated with MIGRATE version 3.6 (Beerli and Felsenstein 1999, 2001). Estimates of migration were obtained for combined loci using the maximum likelihood approach, a full migration matrix model of 10 replicates, four short chains, 100 long‐increments, and 5000 burn‐ins. The first 5000 genealogies in each chain were discarded. Heating was set to be active, with three temperatures (1.0, 1.5, and 3.0) with a swapping interval of 1. All other settings were default to the program. The pairwise models of migration were also tested for a “wrong‐way model” and “panmictic model” where the log‐likelihoods of each model were compared.

### Phylogenetic analysis

Phylogeny was inferred using the Maximum Likelihood method based on the General Time Reversible model (Nei and Kumar 2000) in MEGA 6 (Tamura et al. 2013). The GTR + G + I was determined to be the best model of nucleotide substitution for the data set after a model search based on the highest likelihood score. A discrete Gamma distribution was used to model evolutionary rate differences among sites (6 categories (+*G*, parameter = 4.5930)). The rate variation model allowed for some sites to be evolutionarily invariable ([+*I*], 0.0000% sites). The gene tree with the highest log‐likelihood was shown. The percentage of trees in which the associated taxa clustered together is shown next to the branches. Initial tree(s) for the heuristic search were obtained by using subtree‐pruning‐regrafting (SPR) as the branch‐swapping algorithm. The tree was drawn to scale, with branch lengths measured in the number of substitutions per site. The analysis involved 52 nucleotide sequences. All positions containing gaps and missing data were eliminated. There were a total of 306 positions in the final data set. The robustness of the trees obtained was evaluated by 1000 bootstrap replications. *C. lindemuthianum* was used as the out‐group (Damm et al. 2009). An ML tree was also reconstructed using GARLI (Genetic Algorithm for Rapid Likelihood Inference, http://www.molecularevolution.org/software/phylogenetics/garli). GARLI performs heuristic phylogenetic searches based on the General Time Reversible (GTR) model of nucleotide substitution with or without gamma‐distributed rate heterogeneity and a proportion of invariant sites. The implementation of this model is equivalent to that applied in PAUP*. The ML GARLI tree topology was compared with that of the ML tree generated by MEGA 6.

## Results

### Genetic variation

Table 1(a) and (b) shows the level of DNA polymorphism and sequence variation for both the ITS and *β*‐TUB sequence data sets according to population and for the entire data set. ITS sequences were less informative than the *β*‐TUB sequences based on DNA polymorphism indices. For the ITS sequences, there were only two haplotypes detected, and Hap 1 was the most common, with 45 sequences sharing this haplotype (Table 2). For the *β*‐tubulin sequences, 11 haplotypes were detected, with Hap 2 as the most common, and 34 sequences shared this haplotype. Additional unique haplotypes were found for Trinidad, Thailand, and Malaysia.

**Table 1:** Sequence variation and indices of molecular diversity and neutrality for all populations

	ITS	*β*‐TUB
Sequence variation		
G + C content	0.518	0.484
Total no. of polymorphic loci (S)	1	24
Total no. mutations (Eta)	1	24
Ave. no. nucleotide differences (k)	0.208	2.599
Molecular diversity indices		
Nucleotide diversity (*π*)	0.0005	0.008
Theta S (per sequence)	0.221	5.382
No. of haplotypes (*h*)	2	11
Haplotype diversity (*H*d)	0.208	0.517
Tajima's *D* statistic	−0.079 (*P *≥* *0.100)	−1.689 (*P *≥* *0.100)

**Table 2:** Haplotypes detected for (a) ITS sequences, and (b) β‐TUB sequences

Haplotype designation	No. of haplotypes	Sequences
(a)		
Hap 1	45	[JQ685750| JQ685745| JQ685746| JQ685747| JQ685748| JQ685749| JQ685753| JQ685754| JQ685743| JQ685744| JQ685751| JQ685752| GU227888| GU227886| GU227881| GU227880| GU227877| GQ485593| GU227862| GU227884| GU227875| DQ454028| DQ453990| DQ454026| DQ454024| DQ454013| DQ454027| DQ454017| DQ454025| DQ454014| DQ453989| DQ454016| DQ453987| DQ453988| DQ454015| PEP1| PEP3| PEP5| PEP7| PEP9| PEP2| PEP4| PEP6| PEP8| PEP10]
Hap 2	6	[GU227878| GU227866| GU227865| GU227863| KC110791| KC110790]
(b)		
Hap 1	3	[JX856118| JX856120| JX856121]
Hap 2	34	[JX856119| JX856122| JX856123| JX856124| JX856125| JX856126| JX856127| JX856128| JX856129| GU228182| GU228180| GU228174| GU228172| GU228171| GQ849429| GU228178| GU228160| GU228159| GU228156| KC110818| KC110817| DQ454053| DQ454057| DQ454056| DQ454054| DQ454052| DQ454055| DQ454051| DQ454050| DQ454048| PEP3| PEP5| PEP6| PEP9]
Hap 3	1	[GU228157]
Hap 4	1	[GU228169]
Hap 5	1	[DQ454049]
Hap 6	1	[DQ454046]
Hap 7	1	[DQ454045]
Hap 8	1	[DQ454047]
Hap 9	2	[PEP1| PEP7]
Hap 10	3	[PEP2| PEP4| PEP8]
Hap 11	1	[PEP10]

### Population structure

AMOVA analysis indicated that there was 90% variance within populations and 10% variance between populations (*P *≤* *0.01) based on analysis of combined loci. When individual gene sequences were analyzed, greater genetic variation within populations than among populations was also indicated. The *β*‐TUB gene sequence analysis indicated that 4% of the observed variation was due to differences among populations and 96% of the variation was due to within‐population differences (Table 3). The ITS marker analysis indicated that 26% of the observed variation was due to differences among populations and 74% of the variation was due to within‐population differences.

**Table 3:** Summary of AMOVA statistics: (a) ITS gene, (b) β‐TUB gene, (c) Combined loci

Source	df	SS	MS	Est. Var.	%	*P*‐value
(a)						
Among Populations	4	22.982	5.746	0.175	4%	0.140
Within Populations	46	183.488	3.989	3.989	96%	0.140
Total	50	206.471		4.164	100%	0.140
(b)						
Among Populations	4	39.466	9.866	0.789	26%	0.001
Within Populations	44	99.718	2.266	2.266	74%	0.001
Total	48	139.184		3.055	100%	0.001
(c)						
Among Populations	4	64.914	16.228	0.870	10%	0.010
Within Populations	46	344.655	7.492	7.492	90%	0.010
Total	50	409.569		8.363	100%	0.010

**Figure 1:**
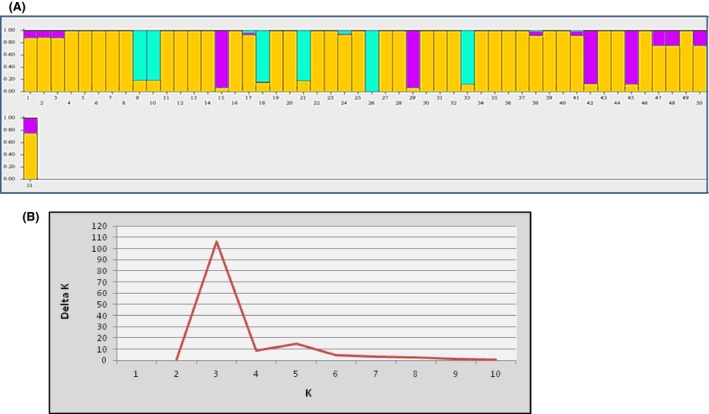
(A) Genetic clustering of individuals belonging to five populations of *C. truncatum* based on 184 polymorphic loci generated from in silico restriction digestion. (B) Plot of delta *K* versus *K* to estimate the most likely value of *K*.

Conservative estimates of population structuring were determined using STRUCTURE. Qualitative understanding of population structure and biological intuition about the relationships were taken into consideration toward settling for a *K* value and which would give the most informative result. *K* = 3 where group 1 (green): Malaysia (#9, #10), India (#18, #20, #26), and USA (#33); group 2 (magenta): Malaysia #15), USA (emia#29), and Trinidad (#42, #45); group 3 (yellow): all Thailand sequences and all other sequences. For this *K* = 3 structuring, all members had a coefficient >80 except for only four members whose coefficient was approximately 20 (Fig. 1A). A plot of delta *K* against *K* also indicated *K* to most likely be 3 (Fig. 1B).

Populations were genetically differentiated despite evidence of part of current gene flow for certain pairwise combinations of populations. The most genetically differentiated populations by pairwise comparisons were India–USA, India–Thailand, India–Malaysia, and India–Trinidad. There was evidence of population differentiation despite evidence of gene flow based on estimates of *β*‐TUB *F*_ST_: The most genetically differentiated populations were USA–Trinidad, Thailand–Trinidad, Malaysia–Trinidad, and India–Trinidad. The Trinidad population was the most differentiated from all other populations in the study. The least differentiated population pairs were USA–Thailand, USA–India, USA–Malaysia, India–Thailand, Malaysia–Thailand, and Malaysia–India (Table 4).

**Table 4:** Wright's *F*_ST_ and Hudson's statistics (*S*_nn_, *H*_s_, and *K*_s_) data for pairwise analysis of populations

Populations	*F*_ST_		*S*_nn_ 1		*H*_s_ 2		*K*_s_ 3	
	ITS	*β*‐TUB	ITS	*β*‐TUB	ITS	*β*‐TUB	ITS	*β*‐TUB
Malaysia–India	0.058	0.075	0.531	0.571	0.095	0.292	0.105	0.273
Malaysia–USA	0.445	0.002	0.742	0.604	0.200	0.447	0.214	0.539
Malaysia–Thailand	NC^4^	0.007	NC	0.559	NC	0.477	NC	0.743
Malaysia–Trinidad	NC	0.084	NC	0.727	NC	0.572	NC	3.303
India–USA	0.096	0.041	0.556	0.500	0.422	0.291	0.419	0.410
India–Thailand	0.006	0.051	0.546	0.531	0.084	0.395	0.095	0.719
India–Trinidad	0.052	0.172	0.501	0.667	0.109	0.519	0.118	4.236
USA–Thailand	0.460	0.016	0.761	0.549	0.179	0.533	0.195	0.950
USA–Trinidad	0.426	0.058	0.722	0.684	0.229	0.680	0.238	4.300
Thailand–Trinidad	NC	0.048	NC	0.700	NC	0.639	NC	3.541

^1^S_nn_ – Nearest‐neighbor statistic (*S*_nn_) is a measure of how often the “nearest neighbors” (in sequence space) sequences are from the same locality in geographic space (Hudson 2000). Values near 1 indicate that two populations at the two localities are highly differentiated. Values near 0.5 indicate that two populations at the two localities are part of the same panmictic population. In cases with small sample sizes (*n*_1_ = *n*_2_ = 10 or 15), especially with recombination, there is substantially higher power with the *S*_nn_ statistic.

^2^ *H*_s_ and ^3^ *K*_s_ statistics ‐sequence‐based statistics found by Hudson et al. (1992). When small sample sizes (*n*_1_ = *n*_2_ = 6) are considered, *K*_S_* and Z* may have slightly higher power than *S*_nn_ when levels of variation are very high.

^4^NC – Not computed because of no polymorphic sites for one or more populations in the pair.

Hudson's statistics (*S*_nn_ *, H*_s_ *,* and *K*_s_) also indicated that all Trinidad population pairings were the most differentiated compared with any other pairwise analyses, regardless of the gene considered.

Tajima's *D* statistic was nonsignificant for both markers and there was no evidence to reject the null hypothesis of neutrality for both gene regions (Table 4).

### Linkage disequilibrium (LD) and recombination

LD and detection of recombination could not be carried out for the populations due to highly invariable ITS sequences with only one polymorphic site detected. Analysis of *β*‐TUB gene sequences indicated that linkage disequilibrium may be present in the sequences under study. Taking only parsimony‐informative sites into account, there were 18 polymorphic loci and 153 pairwise comparisons were considered. There were 115 significant pairwise comparisons according to Fisher's exact test and 15 significant pairwise comparisons based on Bonferroni correction.

Furthermore, based on analyses of 184 polymorphic loci generated from in silico restriction enzyme digests of the ITS and *β*‐TUB sequences of all populations, multilocus linkage disequilibrium tests indicated that both the IA and rBarD statistics were significantly (*P *≤* *0.001) greater than zero, which indicates linkage among loci (Table 5). Using RDP3.44 software, recombination could not be detected in sequences of any population for either the ITS or *β*‐TUB data sets after applying multiple approaches including RDP, GENECONV, MaxChi, and 3Seq methods of analysis.

**Table 5:** Pairwise compatibility, index of association, and rBarD values calculated for combined loci for all populations

Replicate^a^	NumDiff^b^	MaxFreq^c^	Diversity^d^	PrCP^e^	I_A_ f	rBarD^g^
Observed	15	26	0.730	0.953	25.737	0.160
1	48	4	0.995	0.929	1.512	0.009
2	47	2	0.996	0.932	1.587	0.009
3	46	4	0.993	0.928	1.536	0.009
4	48	3	0.996	0.928	1.614	0.010
5	48	3	0.996	0.930	1.520	0.009

a Replicate – Five replicates were used.

b NumDiff – The number of different genotypes.

c MaxFreq – The frequency of the most frequent genotype.

d Diversity – The genotypic diversity, that is, the probability that two individuals taken at random have different genotypes.

e PrCP – The proportion of pairs of loci that are compatible.

f IA – The index of association used to assess if loci are linked.

g rBarD –Accounts for the number of loci sampled that is less biased.

In the association tests, the Index of Association (I_A_) and rBarD are expected to be zero if populations are freely recombining (sexually reproducing) and greater than zero if there is association between alleles (clonality).

### Migration estimates

**Figure 2:**
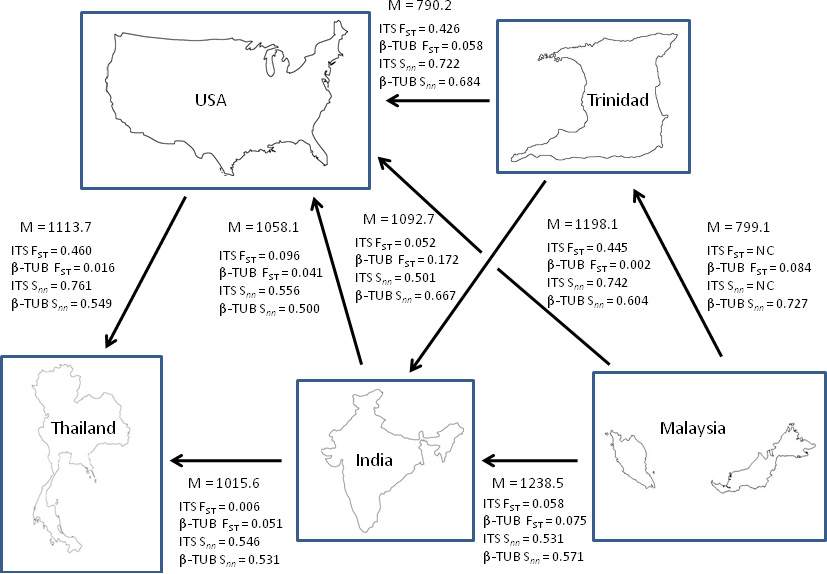
Illustration of the five populations with arrows indicating direction of possible migration events. M is the log‐likelihood of the migration event; Wright's *F*_*ST*_ and Hudson's statistics (*S*_nn_) are also indicated as a measure of genetic differentiation between population pairs.

A model‐based coalescent approach was used to analyze nucleotide sequences (combined loci) to infer migration events that may be occurring among the populations. This approach provides more biologically realistic information, as this type of analysis does not depend on assumptions of populations in equilibrium and of equal size. The direction of gene flow can also be estimated in addition to the rate of migration, taking into account the number of possible migrants involved in the event (Beerli and Fensenstein, 2001). The results provided evidence of asymmetrical migration among populations, which can be explained by historical and/or contemporary anthropogenic events (Table 6a–e). The data suggested one‐way movement of migrants from Malaysia to the four other populations. Migration also occurred from India to Thailand and USA. Movement of the pathogen was traced from Thailand to Trinidad and from USA to Thailand. The results of this study also suggested asymmetrical migration from Trinidad to India and to USA (Fig. 2).

**Table 6:** MIGRATE data for pairwise analysis of combined loci: (a) Posterior distribution table for USA pairwise population analysis, (b) Posterior distribution table for India pairwise population analysis, (c) Posterior distribution table for Thailand pairwise population analysis, (d) Posterior distribution table for Trinidad pairwise population analysis, (e) Posterior distribution table for Malaysia pairwise population analysis

Populations	Parameter	Log‐likelihood						
		2.50%	25.00%	Mode	75.00%	97.50%	Median	Mean
(a)								
M1 = USA	Theta1	0.0000	0.0016	0.0029	0.0042	0.0077	0.0034	54.2410
M2 = Trinidad	Theta2	0.0000	0.0008	0.0031	0.0076	0.0043	0.0072	207.0980
	M2 > 1	128.0000	317.3000	379.0000	688.7000	982.7000	524.3000	790.2000
	M1 > 2	123.3000	251.3000	367.0000	543.3000	910.0000	455.7000	721.5000
M1 = India	Theta1	0.0000	0.0010	0.0023	0.0031	0.0056	0.002370	38.7108
M2 = USA	Theta2	0.0000	0.0013	0.0023	0.0037	0.0138	0.003030	71.1996
	M2 > 1	127.3000	695.3000	980.3000	991.3000	1000.0000	645.0000	909.9000
	M1 > 2	286.0000	744.7000	980.3000	994.7000	1000.0000	751.0000	1058.1000
M1 = USA	Theta1	0.0000	0.0014	0.0026	0.0037	0.0063	0.0029	48.8843
M2 = Thailand	Theta2	0.0003	0.0015	0.0053	0.0207	0.0733	0.0224	509.7152
	M2 > 1	0.0000	44.7000	181.7000	324.7000	791.3000	294.3000	506.4000
	M1 > 2	411.3000	766.0000	981.0000	993.3000	1000.0000	773.0000	1113.7000
M1 = Malaysia	Theta1	0.0000	0.0006	0.0016	0.0023	0.0040	0.0018	26.0494
M2 = USA	Theta2	0.0000	0.0009	0.0036	0.0096	0.0730	0.0090	282.0811
	M2 > 1	0.0000	16.0000	65.7000	209.3000	656.0000	198.3000	370.2000
	M1 > 2	502.7000	828.0000	980.3000	994.7000	1000.0000	834.3000	1198.1000
(b)								
M1 = India	Theta1	0.0000	0.0010	0.0020	0.0031	0.0056	0.0024	38.7108
M2 = USA	Theta2	0.0000	0.0013	0.0025	0.0037	0.0138	0.0030	71.1996
	M2 > 1	127.3000	695.3000	980.3000	991.3000	1000.0000	645.0000	909.9000
	M1 > 2	286.0000	744.7000	980.3000	994.7000	1000.0000	751.0000	1058.1000
M1 = Malaysia	Theta1	0.0000	0.0009	0.0018	0.0027	0.0043	0.0020	29.5712
M2 = India	Theta2	0.0000	0.0000	0.0028	0.0169	0.0825	0.0170	422.1387
	M2 > 1	0.0000	16.0000	75.0000	226.0000	744.7000	214.3000	407.2000
	M1 > 2	546.7000	858.7000	980.3000	996.0000	1000.0000	863.7000	1238.5000
M1 = India	Theta1	0.0000	0.0014	0.0035	0.0063	0.0121	0.0056	384.5368
M2 = Trinidad	Theta2	0.0573	0.0581	0.0600	0.0746	0.0988	0.0713	1050.4876
	M2 > 1	410.0000	730.0000	847.0000	968.7000	1000.0000	755.7000	1092.7000
	M1 > 2	130.7000	276.0000	381.0000	555.3000	890.0000	458.3000	717.4000
M1 = India	Theta1	0.0000	0.0000	0.0026	0.0059	0.0065	0.0644	802.7962
M2 = Thailand	Theta2	0.0000	0.0000	0.0001	0.0010	0.0023	0.0010	15.6565
	M2 > 1	238.0000	527.3000	567.7000	831.3000	1000.0000	643.7000	942.1000
	M1 > 2	360.7000	530.0000	667.7000	792.7000	1000.0000	683.0000	1015.6000
(c)								
M1 = USA	Theta1	0.0000	0.0014	0.0026	0.0037	0.0063	0.0029	48.8843
M2 = Thailand	Theta2	0.0003	0.0015	0.0053	0.0207	0.0733	0.0224	509.7152
	M2 > 1	0.0000	44.7000	181.7000	324.7000	791.3000	294.3000	506.4000
	M1 > 2	411.3000	766.0000	981.0000	993.3000	1000.0000	773.0000	1113.7000
M1 = India	Theta1	0.0000	0.0000	0.0026	0.0059	0.0065	0.0644	802.7962
M2 = Thailand	Theta2	0.0000	0.0000	0.0001	0.0010	0.0023	0.0010	15.6565
	M2 > 1	238.0000	527.3000	567.7000	831.3000	1000.0000	643.7000	942.1000
	M1 > 2	360.7000	530.0000	667.7000	792.7000	1000.0000	683.0000	1015.6000
M1 = Thailand	Theta1	0.0006	0.0034	0.0054	0.0080	0.0167	0.0068	130.9938
M2 = Trinidad	Theta2	0.0000	0.0015	0.0033	0.0078	0.0413	0.0070	173.5854
	M2 > 1	0.0000	17.3000	75.0000	267.3000	752.0000	255.7000	513.3000
	M1 > 2	102.0000	196.7000	303.0000	486.0000	916.0000	417.0000	679.6000
M1 = Malaysia	Theta1	0.0000	0.0009	0.0018	0.0027	0.0041	0.0020	29.0713
M2 = Thailand	Theta2	0.0000	0.0000	0.0000	0.0017	0.0019	0.0364	659.1575
	M2 > 1	0.0000	23.3000	79.0000	202.7000	589.3000	186.3000	342.2000
	M1 > 2	580.0000	859.3000	962.3000	993.3000	1000.0000	867.0000	1250.1000
(d)								
M1 = USA	Theta1	0.0000	0.0016	0.0029	0.0042	0.0077	0.0034	54.2410
M2 = Trinidad	Theta2	0.0000	0.0008	0.0031	0.0076	0.0043	0.0072	207.0980
	M2 > 1	128.0000	317.3000	379.0000	688.7000	982.7000	524.3000	790.2000
	M1 > 2	123.3000	251.3000	367.0000	543.3000	910.0000	455.7000	721.5000
M1 = India	Theta1	0.0000	0.0014	0.0035	0.0063	0.0121	0.0056	384.5368
M2 = Trinidad	Theta2	0.0573	0.0581	0.0600	0.0746	0.0988	0.0713	1050.4876
	M2 > 1	410.0000	730.0000	847.0000	968.7000	1000.0000	755.7000	1092.7000
	M1 > 2	130.7000	276.0000	381.0000	555.3000	890.0000	458.3000	717.4000
M1 = Thailand	Theta1	0.0006	0.0034	0.0054	0.0080	0.0167	0.0068	130.9938
M2 = Trinidad	Theta2	0.0000	0.0015	0.0033	0.0078	0.0413	0.0070	173.5854
	M2 > 1	0.0000	17.3000	75.0000	267.3000	752.0000	255.7000	513.3000
	M1 > 2	102.0000	196.7000	303.0000	486.0000	916.0000	417.0000	679.6000
M1 = Malaysia	Theta1	0.0000	0.0009	0.0019	0.0028	0.0046	0.0022	32.1036
M2 = Trinidad	Theta2	0.0009	0.0052	0.0101	0.0223	0.0685	0.0198	385.8216
	M2 > 1	13.3000	85.3000	199.0000	412.0000	877.3000	360.3000	603.5000
	M1 > 2	172.7000	281.3000	362.3000	593.3000	966.7000	518.3000	799.1000
(e)								
1 = Malaysia	Theta1	0.0000	0.0006	0.0016	0.0023	0.0040	0.0018	26.0494
2 = USA	Theta2	0.0000	0.0009	0.0036	0.0096	0.0730	0.0090	282.0811
	M2 > 1	0.0000	16.0000	65.7000	209.3000	656.0000	198.3000	370.2000
	M1 > 2	502.7000	828.0000	980.3000	994.7000	1000.0000	834.3000	1198.1000
1 = Malaysia	Theta1	0.0000	0.0009	0.0019	0.0028	0.0046	0.0022	32.1036
2 = Trinidad	Theta2	0.0009	0.0052	0.0101	0.0223	0.0685	0.0198	385.8216
	M2 > 1	13.3000	85.3000	199.0000	412.0000	877.3000	360.3000	603.5000
	M1 > 2	172.7000	281.3000	362.3000	593.3000	966.7000	518.3000	799.1000
1 = Malaysia	Theta1	0.0000	0.0009	0.0018	0.0027	0.0041	0.0020	29.0713
2 = Thailand	Theta2	0.0000	0.0000	0.0000	0.0017	0.0019	0.0364	659.1575
	M2 > 1	0.0000	23.3000	79.0000	202.7000	589.3000	186.3000	342.2000
	M1 > 2	580.0000	859.3000	962.3000	993.3000	1000.0000	867.0000	1250.1000
1 = Malaysia	Theta1	0.0000	0.0009	0.0018	0.0027	0.0043	0.0020	29.5712
2 = India	Theta2	0.0000	0.0000	0.0028	0.0169	0.0825	0.0170	422.1387
	M2 > 1	0.0000	16.0000	75.0000	226.0000	744.7000	214.3000	407.2000
	M1 > 2	546.7000	858.7000	980.3000	996.0000	1000.0000	863.7000	1238.5000

### Phylogenetic analysis

**Figure 3:**
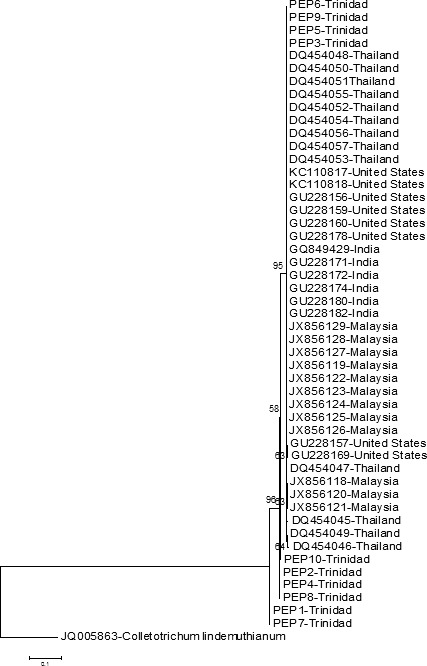
Maximum likelihood tree based on *β*‐TUB gene sequences of *Colletotrichum truncatum*. Bootstrap values above 50% are shown at the nodes. *C. lindemuthianum* is used as the out‐group.

The ML tree based on *β*‐TUB gene sequences revealed three clades, with Clade 1 consisting of all isolates from four populations and only four isolates from Trinidad (96% bootstrap support) (Fig. 3). There was very little subdivision of this clade and short branch lengths were collapsed to polytomies. Clade 2 consisted of four Trinidad isolates with moderate bootstrap support (64%). Clade 3 consisted of the two remaining Trinidad isolates. This third clade appeared to have diverged earlier than the other isolates. A similar tree topology was observed for the ML tree reconstructed with GARLI (tree not shown).

The ML tree based on the ITS sequences was also generated; however, the branching pattern was entirely polytomic, with very poor resolution and low confidence of estimates of isolate placement (tree not shown). This tree reflected the low level of DNA polymorphism of the sequences in the data set, which resulted in very weak phylogenetic signal.

## Discussion

The objectives of the study were (1) to assess the genetic diversity and phylogenetic relationships of *C. truncatum* infecting pepper; (2) to determine the contribution of migration events to the observed genetic variation; (3) to identify the relative contributions of sexual and asexual reproduction to pathogen evolution. The findings presented may only pertain to the set of loci analyzed and may not be representative of patterns that apply to the whole fungal genome. The availability of accurately identified sequences for this fungal species for the two loci under study in the GenBank repository was also a limitation.

The Trinidad population was the most differentiated from all other populations in the study and had the highest nucleotide diversity, which could be explained by (1) secondary contact between divergent lineages or haplotypes resulting in an abundance of and mixing of individuals, (2) high mutation rates, (3) inadvertent sampling of cryptic species, and (4) possible sampling bias for the different populations. There was also evidence of within‐population variation more so than among populations based on AMOVA, and STRUCTURE analyses. High rates of migration and recurrent gene flow as a result of repeated introductions through exchange of infected seed may explain these findings.

The results of this study indicated linkage disequilibrium in the *β*‐TUB gene sequences. There are five processes that can produce linkage disequilibrium in a population: (1) selection, (2) nonrandom mating, (3) genetic drift, (4) genetic hitchhiking, and (5) gene flow (Ridley 2004; Slatkin 2008). Of these five factors, gene flow and selection can produce significant levels of linkage disequilibrium. The data may also indicate asexual reproduction or clonality among the isolates assessed in the study. Sexual reproduction can lead to linkage disequilibrium decay. The teleomorph for *C. truncatum* has never been observed under field conditions, which is common for the *Colletotrichum* genus. *Glomerella truncata* C.L. Armstr. & Banniza was described as the teleomorph of *C. truncatum*, but the strains studied (from lentil in Canada) belong to the destructivum and not to the truncatum clade (Damm et al. 2009). Additionally, which mode of reproduction (sexual or asexual) actually predominates may be influenced by the environment, that is, field ecosystems versus in vitro growth and reproduction, for example, *Phytophthora infestans* and *Magnaporthe oryzae* (Stukenbrock et al., 2006).

The study by Damm et al. (2009) also revealed that *C. truncatum* is pathogenic to different host plants, with the majority from *Fabaceae* and *Capsicum* spp. (Damm et al. 2009). It is hypothesized that because of migration from a *Capsicum*‐infecting population in Trinidad to a non‐*Capsicum* population in the USA, there is little evidence of host discrimination for this plant pathogen. In fact, there were 432 fungus–host combinations for *C. truncatum* in the USDA fungal database (http://nt.ars-grin.gov/fungaldatabases/). Alternatively, the data may suggest a host jump, which has been described for another *Colletotrichum* species (Silva et al. 2012). Further analysis of these two populations is warranted to more conclusively describe the evolutionary history of this fungal pathogen in this region.

There was evidence of asymmetrical migration among populations, which can be explained by historical and/or recent anthropogenic events. We hypothesize two possible mechanisms to explain the asymmetrical movement of the pathogen among the five populations. First, it is proposed that the migration events were recent ones as a result of importation of infected seed. India exports *Capsicum* sp. seeds to USA and Malaysia in addition to China, Japan, Italy, Spain, France, Indonesia, Turkey, Singapore, Sri Lanka, Zimbabwe, South Africa, and the UK; Thailand exported 2037 metric tons valued at US 73 million, India exported 6589 tons valued at US 36 million, and USA exported 17626 tons valued at US 529 million of vegetable seeds including *Capsicum* sp. from 1970 to 2010, according to the International Seed Federation (http://www.worldseed.org/isf/seed_statistics.html).

A second mechanism is proposed to explain asymmetrical migration specifically from Trinidad to India and from Trinidad to USA. Compared with the India population, Trinidad is older, with a higher genetic diversity, and the Trinidad population expanded and diverged earlier. Trinidad also had the larger effective population size in pairwise comparisons and demonstrated significant (*P *≤* *0.05) genetic differentiation from the India population. It is hypothesized that this one‐way migration event to India was an historical one that dated back to the mid‐19th century, as there is no current formalized or direct exportation of any agricultural commodities from Trinidad to India. With the abolition of the Atlantic slave trade in 1833, a system of indentureship or “organized slavery” began in 1845 and ended in 1920 (Williams 1962). In 1844, the Indian government legalized indentured laborers to emigrate from India to Trinidad to work on sugarcane, coffee, cocoa, and coconut estates and during which time, approximately 147,590 Indian laborers came to Trinidad 169 years ago (Tinker 1974). The indentureship agreement included a “free” return passage to India upon successful completion of 5 years' “industrial residence” in the British colony. The last ship carrying returning emigrants left the Caribbean for India in 1954. It is believed that 25% of individuals returned to India (Anonymous, 2014) and anecdotal evidence suggests that repatriated individuals took with them seeds from the island that were not found in India at that time, for example, *Capsicum* seeds. MycoBank repository states that the first reported case of *C. capsici* infection was in 1931 in India by Butler and Bisby in a book entitled “Fungi of India” published by the Government of India, Calcutta (http://www.mycobank.org/BioloMICS.aspx?Link=T&TableKey=14682616000000067&Rec=5901&Fields=All). Compared with the India population, Trinidad may be older, with a higher genetic diversity, and the Trinidad isolates may have diverged earlier. Populations at or near the center of origin of a species have the highest gene diversity (McDonald and Linde 2002). This may explain the directional movement of the pathogen from Trinidad to India as an historical event.

There are no records of pepper export from Trinidad to USA (Mohammed 2013). However, Trinidad is in very close geographic proximity to Mexico, which is the center of origin of *C. annuum*, and it is well documented that free movement of indigenous peoples (the Amerindians of the Caribbean and the Aztecs of Mexico) aided in distributing *Capsicum* throughout the Caribbean basin by, at least, the 16th century (Kraft et al. 2014).The Amerindian populations of North, Central, and South America show a lower genetic diversity than populations from other continental regions (Wang et al. 2007). Low levels of genetic differentiation between Mesoamerican and Andean populations suggest that movement of Paleo‐Indians in these regions was facilitated by coastal routes, for example, the Caribbean Sea, which were easier to cross than inland routes (Wang et al. 2007). This free passage of these indigenous peoples aided in distributing *Capsicum* sp. throughout the Caribbean and possibly to the lower geographic boundaries of North America. Analysis of more protein‐coding gene sequences or whole‐genome sequences of *C. truncatum* would reveal more information concerning population differentiation.

Understanding the genetic variation of pathogen populations, migration potential, and the impact of dual reproductive modes will serve to better inform disease management strategies (McDonald and McDermott 1993; McDonald 1997). Pathogens with high levels of genetic variation and a rapid rate of evolution may be able to adapt to dynamic agro‐ecosystems more successfully than those with lower levels of variation. Such data may be used to predict how long a specific control strategy would be effective (McDonald and Linde 2002).

High recurrent migration rates facilitate rapid movement of new genotypes between populations such that geographic distance among pathogen populations does not necessarily reflect genetic distance. If there is evidence of significant and recurrent gene flow, then it is important to monitor populations for the emergence of new pathotypes that immigrate from distant populations. One approach to achieving long‐term disease control, in such a case, would be to test similar strategies in many geographic locations to gauge the pathogen response in different populations (McDonald and McDermott 1993; McDonald 1997).

According to the risk framework proposed by McDonald and Linde (2002), high genetic diversity and high gene flow, as identified in this study for *C. truncatum* infecting pepper, are indicative of a pathogen with high evolutionary potential. For such pathosystems, control measures that utilize a single protein or biochemical pathway may encourage chemical resistance (McDonald and McDermott 1993; McDonald 1997). Durability of host plant resistance is dependent on several evolutionary factors that impact upon the genetic structure of pathogen populations (McDonald and McDermott 1993). Long‐term, stable control of disease depends on thoughtful integration of these epidemiological and population genetic factors (Milgroom 1996).The findings of this study should lead to a more thorough understanding of natural constraints on pathogen populations and how these can be exploited to develop novel and perhaps shared control strategies (Rampersad et al. 2013).

## Conflict of Interest

The authors declare no conflict of interest.

## Data accessibility

Supplementary Table S1 (A) and (B) can be found in the Dryad public archive using the following link below: http://dx.doi.org/10.5061/dryad.353gc.

## Supporting information


**Table S1.** (A). ITS sequences of the five populations used in the study. (B) β‐TUB sequences of the five populations used in the study.
