# Supplementary material for: Utility of internally transcribed spacer region of rDNA (ITS) and β‐tubulin gene sequences to infer genetic diversity and migration patterns of Colletotrichum truncatum infecting Capsicum spp
Source: Ecol Evol. 2016 Jan 9;6(2):593–606. doi: 10.1002/ece3.1918 (PMC4729253; doi:10.1002/ece3.1918)
Supplement: Supplementary file 1 — Table S1. (A). ITS sequences of the five populations used in the study. (B) β‐TUB sequences of the five populations used in the study. [file ECE3-6-593-s001.docx]

**Supplementary Table S1 (a). ITS sequences of the five populations used in the study**

| **GenBank Accession No.** | **Strain** | **Country** | **Host** | **Reference** |
| --- | --- | --- | --- | --- |
| [JQ685754](https://www.ncbi.nlm.nih.gov/nuccore/JQ685754.1) | CCM10 | Malaysia | *Capsicum* spp. | Mahmodi et al. 2014 |
| [JQ685753](https://www.ncbi.nlm.nih.gov/nuccore/JQ685753.1) | CCM9 | Malaysia | *Capsicum* spp. | Mahmodi et al. 2014 |
| [JQ685752](https://www.ncbi.nlm.nih.gov/nuccore/JQ685752.1) | CCM6 | Malaysia | *Capsicum* spp. | Mahmodi et al. 2014 |
| [JQ685751](https://www.ncbi.nlm.nih.gov/nuccore/JQ685751.1) | CCM5 | Malaysia | *Capsicum* spp. | Mahmodi et al. 2014 |
| [JQ685750](https://www.ncbi.nlm.nih.gov/nuccore/JQ685750.1) | CCM2 | Malaysia | *Capsicum* spp. | Mahmodi et al. 2014 |
| [JQ685744](https://www.ncbi.nlm.nih.gov/nuccore/JQ685744.1) | CCM12 | Malaysia | *Capsicum* spp. | Mahmodi et al. 2014 |
| [JQ685743](https://www.ncbi.nlm.nih.gov/nuccore/JQ685743.1) | CCM11 | Malaysia | *Capsicum* spp. | Mahmodi et al. 2014 |
| [JQ685748](https://www.ncbi.nlm.nih.gov/nuccore/JQ685748.1) | CCM7 | Malaysia | *Capsicum* spp. | Mahmodi et al. 2014 |
| [JQ685749](https://www.ncbi.nlm.nih.gov/nuccore/JQ685749.1) | CCM8 | Malaysia | *Capsicum* spp. | Mahmodi et al. 2014 |
| [JQ685747](https://www.ncbi.nlm.nih.gov/nuccore/JQ685747.1) | CCM4 | Malaysia | *Capsicum* spp. | Mahmodi et al. 2014 |
| [JQ685745](https://www.ncbi.nlm.nih.gov/nuccore/JQ685745.1) | CCM1 | Malaysia | *Capsicum* spp. | Mahmodi et al. 2014 |
| [DQ454028](https://www.ncbi.nlm.nih.gov/nuccore/DQ454028.1) | U12 | Thailand | *Capsicum annuum* | Than et al. 2008 |
| [DQ454027](https://www.ncbi.nlm.nih.gov/nuccore/DQ454027.1) | U10 | Thailand | *Capsicum annuum* | Than et al. 2008 |
| [DQ454026](https://www.ncbi.nlm.nih.gov/nuccore/DQ454026.1) | U9 | Thailand | *Capsicum annuum* | Than et al. 2008 |
| [DQ454025](https://www.ncbi.nlm.nih.gov/nuccore/DQ454025.1) | Skp16 | Thailand | *Capsicum annuum* | Than et al. 2008 |
| [DQ454024](https://www.ncbi.nlm.nih.gov/nuccore/DQ454024.1) | Skp4 | Thailand | *Capsicum annuum* | Than et al. 2008 |
| [DQ454017](https://www.ncbi.nlm.nih.gov/nuccore/DQ454017.1) | R12 | Thailand | *Capsicum annuum* | Than et al. 2008 |
| [DQ454016](https://www.ncbi.nlm.nih.gov/nuccore/DQ454016.1) | R11 | Thailand | *Capsicum annuum* | Than et al. 2008 |
| [DQ454015](https://www.ncbi.nlm.nih.gov/nuccore/DQ454015.1) | R7 | Thailand | *Capsicum annuum* | Than et al. 2008 |
| [DQ454014](https://www.ncbi.nlm.nih.gov/nuccore/DQ454014.1) | R5 | Thailand | *Capsicum annuum* | Than et al. 2008 |
| [DQ454013](https://www.ncbi.nlm.nih.gov/nuccore/DQ454013.1) | R4 | Thailand | *Capsicum annuum* | Than et al. 2008 |
| [DQ453990](https://www.ncbi.nlm.nih.gov/nuccore/DQ453990.1) | Ccmj10 | Thailand | *Capsicum annuum* | Than et al. 2008 |
| [DQ453989](https://www.ncbi.nlm.nih.gov/nuccore/DQ453989.1) | Ccmj7 | Thailand | *Capsicum annuum* | Than et al. 2008 |
| [DQ453988](https://www.ncbi.nlm.nih.gov/nuccore/DQ453988.1) | Ccmj3 | Thailand | *Capsicum annuum* | Than et al. 2008 |
| [DQ453987](https://www.ncbi.nlm.nih.gov/nuccore/DQ453987.1) | Ccmj2 | Thailand | *Capsicum annuum* | Than et al. 2008 |
| [GU227866](https://www.ncbi.nlm.nih.gov/nuccore/GU227866.1) | CBS:182.52 | USA | *Glycine max* | Damm et al., 2009 |
| [GU227865](https://www.ncbi.nlm.nih.gov/nuccore/GU227865.1) | CBS:195.32 | USA | *Glycine max* | Damm et al., 2009 |
| [GU227863](https://www.ncbi.nlm.nih.gov/nuccore/GU227863.1) | CBS:119189 | USA | *Phaseolus lunatus* | Damm et al., 2009 |
| [GU227862](https://www.ncbi.nlm.nih.gov/nuccore/GU227862.1) | CBS:151.35 | USA | *Phaseolus lunatus* | Damm et al., 2009 |
| [GU227884](https://www.ncbi.nlm.nih.gov/nuccore/GU227884.1) | CBS:146.32 | USA | *Opuntia sp.* | Damm et al., 2009 |
| GU227875 | CBS:260.85 | USA | *Crotalaria spectabilis* | Damm et al., 2009 |
| [KC110791](https://www.ncbi.nlm.nih.gov/nuccore/KC110791.1) | IL16D | USA | *Glycine max* | Yang et al., 2010 |
| [KC110790](https://www.ncbi.nlm.nih.gov/nuccore/KC110790.1) | IL15B | USA | *Glycine max* | Yang et al., 2010 |
| [GU227888](https://www.ncbi.nlm.nih.gov/nuccore/GU227888.1) | CBS:127.57 | India | *Peperomia magnoliifolia* | Damm et al., 2009 |
| [GU227886](https://www.ncbi.nlm.nih.gov/nuccore/GU227886.1) | IMI:63597 | India | *Peperomia magnoliifolia* | Damm et al., 2009 |
| [GU227880](https://www.ncbi.nlm.nih.gov/nuccore/GU227880.1) | CBS:371.67 | India | *Capsicum annuum* | Damm et al., 2009 |
| [GU227877](https://www.ncbi.nlm.nih.gov/nuccore/GU227877.1) | CBS:120709 | India | *Capsicum frutescens* | Damm et al., 2009 |
| [GQ485593](https://www.ncbi.nlm.nih.gov/nuccore/GQ485593.1) | CBS 120709 | India | *Capsicum frutescens* | Yang et al., 2010 |
| [JF749808](http://www.ncbi.nlm.nih.gov/nuccore/JF749808) | PEPcc-001 | Trinidad | *Capsicum annuum* | Ramdial and Rampersad, 2011 |
|  | PEP11 | Trinidad | *Capsicum annuum* | This study |
| [KJ780718](http://www.ncbi.nlm.nih.gov/nuccore/?term=kj780718) | PEP10/14 | Trinidad | *Capsicum annuum* | 2014 |
|  | PEP3 | Trinidad | *Capsicum annuum* | This study |
|  | PEP7 | Trinidad | *Capsicum annuum* | This study |
|  | PEP9 | Trinidad | *Capsicum annuum* | This study |
|  | PEP2 | Trinidad | *Capsicum annuum* | This study |
|  | PEP4 | Trinidad | *Capsicum annuum* | This study |
|  | PEP6 | Trinidad | *Capsicum annuum* | This study |
|  | PEP8 | Trinidad | *Capsicum annuum* | This study |

**Supplementary Table S1 (b). β-TUB sequences of the five populations used in the study**

| **GenBank Accession No.** | **Strain** | **Country** | **Host** | **Reference** |
| --- | --- | --- | --- | --- |
| [JX856129](https://www.ncbi.nlm.nih.gov/nuccore/JX856129.1) | CCM12 | Malaysia | *Capsicum* spp. | Mahmodi et al. 2014 |
| [JX856128](https://www.ncbi.nlm.nih.gov/nuccore/JX856128.1) | CCM11 | Malaysia | *Capsicum* spp. | Mahmodi et al. 2014 |
| [JX856127](https://www.ncbi.nlm.nih.gov/nuccore/JX856127.1) | CCM10 | Malaysia | *Capsicum* spp. | Mahmodi et al. 2014 |
| [JX856126](https://www.ncbi.nlm.nih.gov/nuccore/JX856126.1) | CCM9 | Malaysia | *Capsicum* spp. | Mahmodi et al. 2014 |
| [JX856125](https://www.ncbi.nlm.nih.gov/nuccore/JX856125.1) | CCM8 | Malaysia | *Capsicum* spp. | Mahmodi et al. 2014 |
| [JX856124](https://www.ncbi.nlm.nih.gov/nuccore/JX856124.1) | CCM7 | Malaysia | *Capsicum* spp. | Mahmodi et al. 2014 |
| [JX856123](https://www.ncbi.nlm.nih.gov/nuccore/JX856123.1) | CCM6 | Malaysia | *Capsicum* spp. | Mahmodi et al. 2014 |
| [JX856122](https://www.ncbi.nlm.nih.gov/nuccore/JX856122.1) | CCM5 | Malaysia | *Capsicum* spp. | Mahmodi et al. 2014 |
| [JX856121](https://www.ncbi.nlm.nih.gov/nuccore/JX856121.1) | CCM4 | Malaysia | *Capsicum* spp. | Mahmodi et al. 2014 |
| [JX856120](https://www.ncbi.nlm.nih.gov/nuccore/JX856120.1) | CCM3 | Malaysia | *Capsicum* spp. | Mahmodi et al. 2014 |
| [JX856119](https://www.ncbi.nlm.nih.gov/nuccore/JX856119.1) | CCM2 | Malaysia | *Capsicum* spp. | Mahmodi et al. 2014 |
| [JX856118](https://www.ncbi.nlm.nih.gov/nuccore/JX856118.1) | CCM1 | Malaysia | *Capsicum annuum* | Than et al. 2008 |
| [DQ454057](https://www.ncbi.nlm.nih.gov/nuccore/DQ454057.1) | R12 | Thailand | *Capsicum annuum* | Than et al. 2008 |
| [DQ454056](https://www.ncbi.nlm.nih.gov/nuccore/DQ454056.1) | R5 | Thailand | *Capsicum annuum* | Than et al. 2008 |
| [DQ454055](https://www.ncbi.nlm.nih.gov/nuccore/DQ454055.1) | Skp16 | Thailand | *Capsicum annuum* | Than et al. 2008 |
| [DQ454054](https://www.ncbi.nlm.nih.gov/nuccore/DQ454054.1) | Ccmj10 | Thailand | *Capsicum annuum* | Than et al. 2008 |
| [DQ454053](https://www.ncbi.nlm.nih.gov/nuccore/DQ454053.1) | R7 | Thailand | *Capsicum annuum* | Than et al. 2008 |
| [DQ454052](https://www.ncbi.nlm.nih.gov/nuccore/DQ454052.1) | Skp4 | Thailand | *Capsicum annuum* | Than et al. 2008 |
| [DQ454051](https://www.ncbi.nlm.nih.gov/nuccore/DQ454051.1) | U12 | Thailand | *Capsicum annuum* | Than et al. 2008 |
| [DQ454050](https://www.ncbi.nlm.nih.gov/nuccore/DQ454050.1) | U10 | Thailand | *Capsicum annuum* | Than et al. 2008 |
| [DQ454049](https://www.ncbi.nlm.nih.gov/nuccore/DQ454049.1) | R11 | Thailand | *Capsicum annuum* | Than et al. 2008 |
| [DQ454048](https://www.ncbi.nlm.nih.gov/nuccore/DQ454048.1) | Ccmj7 | Thailand | *Capsicum annuum* | Than et al. 2008 |
| [DQ454047](https://www.ncbi.nlm.nih.gov/nuccore/DQ454047.1) | Ccmj3 | Thailand | *Capsicum annuum* | Than et al. 2008 |
| [DQ454046](https://www.ncbi.nlm.nih.gov/nuccore/DQ454046.1) | R4 | Thailand | *Capsicum annuum* | Than et al. 2008 |
| [DQ454045](https://www.ncbi.nlm.nih.gov/nuccore/DQ454045.1) | Ccmj2 | Thailand | *Capsicum annuum* | Than et al. 2008 |
| [GU228178](https://www.ncbi.nlm.nih.gov/nuccore/GU228178.1) | CBS:146.32 | USA | *Glycine max* | Damm et al., 2009 |
| [GU228169](https://www.ncbi.nlm.nih.gov/nuccore/GU228169.1) | CBS:260.85 | USA | *Glycine max* | Damm et al., 2009 |
| [GU228160](https://www.ncbi.nlm.nih.gov/nuccore/GU228160.1) | CBS:182.52 | USA | *Phaseolus lunatus* | Damm et al., 2009 |
| [GU228159](https://www.ncbi.nlm.nih.gov/nuccore/GU228159.1) | CBS:195.32 | USA | *Phaseolus lunatus* | Damm et al., 2009 |
| [GU228157](https://www.ncbi.nlm.nih.gov/nuccore/GU228157.1) | CBS:119189 | USA | *Opuntia sp.* | Damm et al., 2009 |
| [GU228156](https://www.ncbi.nlm.nih.gov/nuccore/GU228156.1) | CBS:151.35 | USA | *Crotalaria spectabilis* | Damm et al., 2009 |
| [KC110818](https://www.ncbi.nlm.nih.gov/nuccore/KC110818.1) | IL16D | USA | *Glycine max* | Yang et al., 2010 |
| [KC110817](https://www.ncbi.nlm.nih.gov/nuccore/KC110817.1) | IL15B | USA | *Glycine max* | Yang et al., 2010 |
| [GU228182](https://www.ncbi.nlm.nih.gov/nuccore/GU228182.1) | CBS:127.57 | India | *Peperomia magnoliifolia* | Damm et al., 2009 |
| [GU228180](https://www.ncbi.nlm.nih.gov/nuccore/GU228180.1) | IMI:63597 | India | *Peperomia magnoliifolia* | Damm et al., 2009 |
| [GU228174](https://www.ncbi.nlm.nih.gov/nuccore/GU228174.1) | CBS:371.67 | India | *Capsicum annuum* | Damm et al., 2009 |
| [GU228171](https://www.ncbi.nlm.nih.gov/nuccore/GU228171.1) | CBS:120709 | India | *Capsicum frutescens* | Yang et al., 2010 |
| [GQ849429](https://www.ncbi.nlm.nih.gov/nuccore/GQ849429.1) | CBS 120709 | India | *Capsicum frutescens* | Yang et al., 2010 |
| [JF749808](http://www.ncbi.nlm.nih.gov/nuccore/JF749808) | PEP1 | Trinidad | *Capsicum annuum* | Ramdial and Rampersad, 2014 |
|  | PEP3 | Trinidad | *Capsicum annuum* | This study |
|  | PEP15 | Trinidad | *Capsicum annuum* | This study |
|  | PEP7 | Trinidad | *Capsicum annuum* | This study |
|  | PEP9 | Trinidad | *Capsicum annuum* | This study |
|  | PEP2 | Trinidad | *Capsicum annuum* | This study |
|  | PEP4 | Trinidad | *Capsicum annuum* | This study |
|  | PEP6 | Trinidad | *Capsicum annuum* | This study |
|  | PEP8 | Trinidad | *Capsicum annuum* | This study |
| [HQ287585.1](http://www.ncbi.nlm.nih.gov/nuccore/HQ287585.1) | PEP10 | Trinidad | *Capsicum annuum* | 2010 |
